# Do Family Physicians’ Recommendations for Influenza and Pneumococcal Vaccines Impact the Elderly Aged ≥60 Years? A Cross-Sectional Study in Six Chinese Cities

**DOI:** 10.3390/vaccines13080886

**Published:** 2025-08-21

**Authors:** Yuxing Wang, Jianing Dai, Shuai Yuan, Ying Chen, Zhujiazi Zhang, Ling Zhu, Gang Liu, Qiang Zeng, Qian Qiu, Chunyu Luo, Rendan Deng, Lili You

**Affiliations:** 1 School of Health Policy and Management, Chinese Academy of Medical Sciences and Peking Union Medical College, Beijing 100080, China; wangyx@student.pumc.edu.cn (Y.W.); daijn@pumc.edu.cn (J.D.); chenying@pumc.edu.cn (Y.C.); 2 Department of Health Policy and Management, Bloomberg School of Public Health, Johns Hopkins University, Baltimore, MD 21205, USA; shuai.yuan@jhu.edu; 3 Institute for Hospital Management, Tsinghua University, Shenzhen 518000, China; 4 Beijing Center for Disease Prevention and Control, Beijing 100013, China; 2411121003@stu.pku.edu.cn (Z.Z.); qiuq@bjcdc.org (Q.Q.); 5 Nanan District Center for Disease Control and Prevention, Chongqing 400060, China; 13650502603@163.com (L.Z.); chunyul2025@163.com (C.L.); rendand2025@163.com (R.D.); 6 Shenzhen Center for Disease Control and Prevention, Shenzhen 518055, China; sliugang@163.com; 7 Shenzhen Nanshan District Health Committee, Shenzhen 518052, China; email_zeng@163.com

**Keywords:** family physician recommendation, influenza vaccine, PPSV-23, urban elderly in China, cross-sectional study, marginal effects

## Abstract

**Background**: Influenza vaccine and pneumococcal vaccine are essential to protect the health of older adults. This study focuses on the impact of family physicians’ recommendations on influenza and pneumococcal vaccine uptake among urban Chinese older adults and makes recommendations for improving vaccination rates. **Methods**: A cross-sectional survey on influenza vaccination and pneumonia vaccination was conducted in December 2024 in six cities in China among adults aged ≥60 years. Marginal effects as well as logistic regression models were adopted to measure the relationship between family physician recommendation and influenza vaccination and pneumonia vaccination. **Results**: The overall influenza vaccination rate was 34.05% and pneumococcal vaccination rate was 22.79%. City, educational level, monthly income, health status, and family physician vaccination recommendation had significant impacts on influenza and pneumococcal vaccination (*p* < 0.05). Among the investigated elderly population, 48.78% and 28.56% had received recommendations from family physicians regarding influenza and pneumococcal vaccination, respectively. Analysis of marginal effects models revealed that physicians’ recommendations were significantly able to boost influenza and pneumococcal vaccination rates by 26.3% (average marginal effect = 0.263, 95% CI = 0.249–0.277) and 23.7% (average marginal effect = 0.237, 95% CI = 0.225–0.248), respectively (*p* < 0.001). In the adjusted model, family physician recommendation, compared with no family physician recommendation, was also associated with vaccine policy, monthly income, and age in influenza vaccine and pneumococcal vaccine uptake. **Conclusions**: Older adults’ influenza and pneumococcal vaccination rates need to be improved. Family physicians’ recommendations show a more significant impact on older adults. Family physician recommendations had the greatest boosting effect on vaccination among individuals aged 70–79. Healthcare providers should adopt different vaccine recommendation strategies based on the characteristics of older adults.

## 1. Introduction

Due to lower immune responses associated with aging, influenza and pneumococcal disease are linked to considerable morbidity and mortality in older adults [1,2,3]. Based on an estimate, influenza causes approximately 290,000–650,000 deaths annually, while pneumonia causes 2,600,000 deaths [4,5,6]. Among Chinese older adults, influenza and pneumonia also pose a serious health threat [7,8]. Preventing influenza and pneumonia is crucial for the health development of the population.

Influenza and pneumococcal vaccination are the most effective ways to prevent morbidity and mortality attributable to pneumococcal pneumonia and influenza virus, as well as to reduce economic burdens [9]. According to the World Health Organization (WHO), older adults should receive routine immunization with influenza and pneumococcal vaccines, and many countries currently follow this recommendation [10,11,12,13,14]. In China, the Chinese Center for Disease Control and Prevention (CDC) guidelines recommend influenza and pneumococcal vaccination for populations at high risk of infection and influenza-related complications among older adults [15,16]. Meanwhile, the policy of subsidizing influenza vaccines and pneumonia vaccines has been implemented in some Chinese cities with sufficient financial budgets, like Beijing and Shenzhen [17,18,19,20].

Past research has shown that factors such as age, education level, health status, and economic status influence vaccination rates [7,21,22,23]. In addition, prior studies have indicated that provider-level barriers among populations contribute to the low vaccination coverage in China [23,24]. Since the implementation of the family physician contract service, family physicians have provided health education services to elderly people in the community and have become the medical professionals most frequently encountered by the elderly [25]. In China, family physicians include physicians in primary healthcare institutions like Community Health Service Centers (CHSCs), as well as qualified physicians from township health centers, rural doctors, and physicians in the Chinese medicine category [25]. A family physician’s vaccination recommendation can directly affect older adults’ trust in vaccines and subsequent vaccination decisions. After receiving vaccination recommendations from their physicians, older adults are more motivated to get vaccinated and have increased confidence in the safety and efficacy of vaccines [17]. The vaccination status of primary care physicians and patients’ mistrust toward physicians are important factors influencing recipients’ willingness to be vaccinated [26,27]. Notably, among the elderly population, those with comorbid chronic conditions—such as hypertension, diabetes, or cardiovascular diseases—exhibit a higher propensity to follow medical advice regarding vaccinations [28]. Lack of recommendation by family physicians has been cited as one of the main reasons why pneumococcal and influenza vaccinations were rejected in previous studies. Moreover, the current involvement of general practitioners in vaccination recommendations is low in China. Few family physicians actively provide vaccination recommendations for high-risk groups such as the elderly in the past [29,30]. Current academic inquiry into the role of family physician recommendations in driving influenza and pneumonia vaccination uptake among older adults remains conspicuously underdeveloped.

This study aims to investigate the relationship between physicians’ recommendations and influenza and pneumococcal vaccination among older adults in China. Findings from this study can provide valuable insights into health policies on influenza and pneumococcal vaccination among older adults in China and contribute to efforts to improve vaccination coverage.

## 2. Method

### 2.1. Study Design and Participants

**Figure 1:**
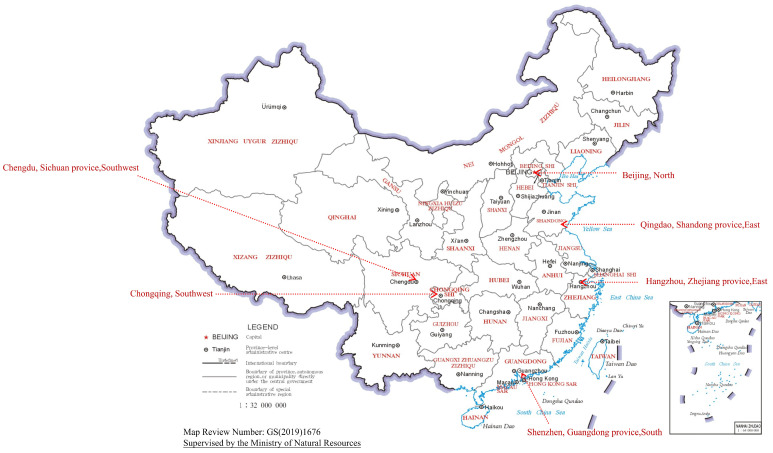
Distribution of sampled participants.

**Figure 2:**
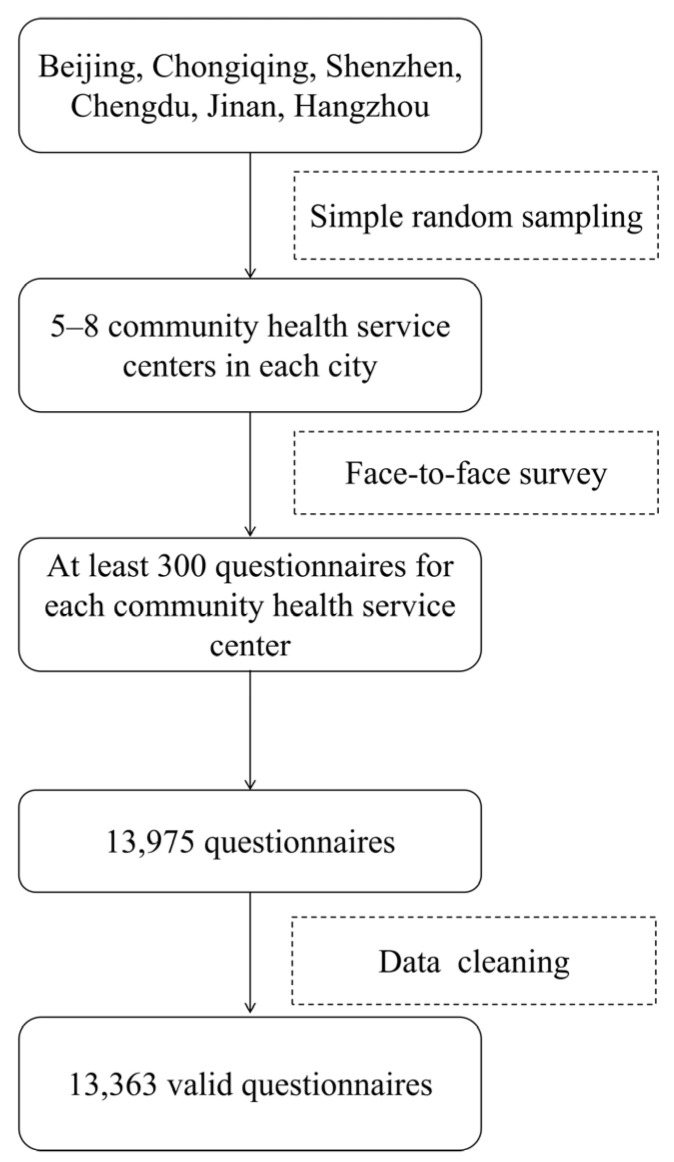
Recruitment process of sampled participants.

In December 2024, we conducted a cross-sectional study to collect self-reported data through field surveys in Beijing (North), Hangzhou (East), Qingdao (East), Shenzhen (South), Chongqing (Southwest), and Chengdu (Southwest), which were selected based on geographic location. In each city, five to eight Community Health Service Centers (CHSCs)—responsible for providing vaccination services to urban residents—were randomly selected using simple random sampling from a list of all local CHSCs. Each CHSCs needed to invite 300 individuals. The details can be seen in Figure 1 and Figure 2.

Elderly individuals aged 60 years and above who received free medical examinations at the CHSCs were randomly invited to participate and were surveyed when they came for health checkups or vaccinations. Interviewers approached eligible individuals consecutively in waiting areas and invited them to participate. All questionnaires were completed through face-to-face interviews conducted by trained interviewers and were configured to require completion of all items before submission. In total, 13,754 individuals were invited and completed the questionnaire. During the data processing stage, we applied strict inclusion criteria and logical consistency checks to screen the collected questionnaires and remove invalid samples that did not meet the age threshold or contained contradictory responses, thereby ensuring the validity of the dataset. A total of 13,363 valid questionnaires were ultimately included, resulting in a response validity rate of 97.2%.

### 2.2. Measures

The primary outcome variables were binary indicators of whether the participant had ever received the influenza vaccine and pneumococcal vaccine. Participants were asked: “Have you received influenza vaccine for adults in the past one year?” and “Have you ever received pneumococcal vaccine for adults?”. All dependent variables were binary, with response categories of “Yes” or “No”.

The key explanatory variable was family physicians’ recommendations. Participants were asked: “Has a family physician ever recommended that you receive the influenza vaccine?” and “Has a family physician ever recommended that you receive the pneumococcal vaccination?”. Responses were binary, with the response categories as “Yes” or “No”. If participants answered “No”, we classified as “no-family physician recommendation.”

The questionnaire also collected demographic characteristics and health-related information, including gender, age, ethnicity, education level, monthly income, self-reported health status, and whether the vaccine was free or self-paid. Details of the questionnaire are provided in Supplementary Material (File S1).

### 2.3. Statistical Analysis

Statistical analyses were performed using SPSS Version 25.0 and R Version 4.4.3. First, we estimated vaccination rates for both vaccines among older adults, as well as rates for each vaccine based on sociodemographic and health-related characteristics of the participants. A chi-square test was performed to discern differences between groups.

We then assessed the association between influenza vaccination and family physician recommendation status using an average marginal effect (AME), controlling for age category, gender, race, education level, monthly income, health status, and vaccine price. To conduct the sensitivity analysis, 50% of the sample size was repeatedly drawn from the original dataset for 200 iterations. The range of AME values was calculated, and the original AME value was compared against this range to verify its inclusion, thereby completing the analysis.

Finally, we tested for differences in vaccination rates associated with age/vaccine price/monthly income and family physician recommendation by two logistic regressions. We used R’s marginaleffects commands to calculate predicted probabilities from each model (note that estimates of unadjusted probabilities using this approach are identical to simple cross-tabulations). We then calculated the marginal effect on influenza and pneumococcal vaccination within each of the three population categories, for each age category, and for each lower income group versus the highest income category. Using estimates from our adjusted effect modification models, we calculated the differential effect of being 70–79 and ≥80 years old versus 60–69 years old, female versus male, or low and middle income versus high income among those without a family physician recommendation relative to that difference in the elderly who had received a recommendation from a family physician.

## 3. Result

### 3.1. Population Characteristics and Factors Associated with Vaccination

Table 1 displays the basic characteristics of 13,363 participants among six cities in China. Of these, 45.26% were male, and most participants were aged 60–69 years (57.46%). Additionally, 43.62% of participants reported a monthly income of CNY 2501 to 5000. Most participants rated their health status as fair (40.16%) or good (40.57%). A total of 34.05% of participants received the influenza vaccine free of charge, while 22.79% received the pneumococcal vaccine free of charge. The overall influenza vaccination rate was 34.05% and pneumococcal vaccination rate was 22.79%.

City (χ^2^ = 187.684, *p* < 0.001), educational level (χ^2^ = 99.695, *p* < 0.001), monthly income (χ^2^ = 157.152, *p* < 0.001), health status (χ^2^ = 15.594, *p* = 0.004), and family physician vaccination recommendation (χ^2^ = 1058.091, *p* < 0.001) all had significant impacts on influenza vaccination. Similarly, city (χ^2^ = 271.66, *p* < 0.001), educational level (χ^2^ = 59.038, *p* < 0.001), monthly income (χ^2^ = 36.476, *p* < 0.001), health status (χ^2^ = 52.851, *p* < 0.001), and family physician vaccination recommendation (χ^2^ = 1251.479, *p* < 0.001) significantly impacted pneumococcal vaccination. For details, see Table 2.

### 3.2. Analysis of Marginal Effects of Family Physicians’ Recommendations

The AME value for family physicians giving influenza vaccination recommendations was 0.263, suggesting that physicians’ vaccine recommendations were able to boost influenza vaccination rates by 26.3% (95% CI = 0.249–0.277). Through 200 random samples of 50% of the data, the AME value was found to be stably distributed within the range of 0.250 to 0.275, indicating that the results are insensitive to data disturbances and exhibit good robustness.

The AME value for physicians giving pneumococcal vaccination recommendation was 0.237, suggesting that physicians’ pneumococcal vaccination recommendation was able to boost vaccination rates by 23.7% (95% CI = 0.225–0.248). Through 200 random samples of 50% of the data, the AME value was found to be stably distributed within the range of 0.227 to 0.250, indicating that the results are insensitive to data disturbances and exhibit good robustness.

### 3.3. Vaccine Policy Differences in the Probability of Influenza Vaccine and Pneumococcal Vaccine with Family Physician’s Recommendation and No Family Physician’s Recommendation

Table 3 provides unadjusted and adjusted rates of influenza and pneumococcal vaccine by vaccination price. For the influenza vaccine, among participants without a family physician recommendation, the adjusted probability for free vaccination (21.53%) was 1.64% (95% CI = 0.002–0.031) higher than for self-paid vaccination (19.89%). Among those with family physician recommendation, the adjusted probability for free vaccination (49.86%) was 2.50% (95% CI = 0.027–0.047) higher than for self-paid vaccination (47.36%). For the effect of family physician recommendation by vaccine price, the adjusted probability of vaccination with family physician recommendation was 27.47% (95% CI = 0.259–0.290) and 28.33% (95% CI = 0.265–0.301) higher, respectively, than the probability of vaccination without a recommendation for the self-paid and free vaccine groups. For pneumococcal vaccine, the effect of vaccine price by family physician recommendation was non-significant in all analyses. However, regarding the effect of family physician recommendation by vaccine price, the adjusted probability for self-paid vaccination was 28.78% (95% CI = 0.266–0.304) and for free vaccination was 28.91% (95% CI = 0.267–0.305) higher with family physician recommendation than without.

### 3.4. Age-Group Differences in the Probability of Influenza Vaccine and Pneumococcal Vaccine with Family Physician Recommendation and No Family Physician Recommendation

Table 4 provides unadjusted and adjusted rates of influenza and pneumococcal vaccination by age group. For influenza vaccination, the effect of age by family physician recommendation was non-significant in all analyses. However, older individuals with a physician’s recommendation had a higher probability of receiving both the influenza and pneumococcal vaccines compared to those without a physician’s recommendation, and this difference was significant in all analyses. Individuals aged 70–79 had the highest probability of vaccination in all analyses for both influenza and pneumococcal vaccines. For instance, in pneumococcal vaccination among those without a physician’s recommendation, the probability for individuals aged 70–79 was 15.41%, higher than for those aged 60–69 (14.12%) and those aged ≥80 (13.93%).

### 3.5. Gender Differences in the Probability of Influenza Vaccine and Pneumococcal Vaccine with Family Physician Recommendation and No Family Physician Recommendation

Table 5 provides unadjusted and adjusted rates of influenza and pneumococcal vaccination by gender. The result shows that participants with a family physician’s recommendation had a higher probability of vaccination than those without a recommendation for both influenza and pneumococcal vaccines, with significant differences in all analyses. For instance, in influenza vaccine, among males, the effect of a family physician recommendation on vaccination probability was 27.39% (95% CI = 0.262, 0.296).

### 3.6. Monthly Income Differences in the Probability of Influenza Vaccine and Pneumococcal Vaccine with Family Physician Recommendation and No Family Physician Recommendation

Table 6 provides unadjusted and adjusted rates of influenza and pneumococcal vaccination by monthly income. The result shows that older persons with high monthly incomes had a higher probability of receiving both the influenza and pneumococcal vaccines compared to those with lower monthly incomes, and these differences were significant in all analyses. For instance, for the influenza vaccine, among those without a physician’s recommendation, the adjusted probability for individuals with a monthly income of CNY 2500–5000 (21.37%) was 5.54% (95% CI = 0.041–0.070) higher than for those with a monthly income below CNY 2500 (15.83%). In addition, participants with a family physician’s recommendation had a higher probability of vaccination than those without a recommendation for both the influenza and pneumococcal vaccines, with significant differences in all analyses. For instance, for the pneumococcal vaccine, among individuals with a monthly income of CNY 5000–7500, the effect of a family physician’s recommendation on vaccination probability was 30.46% (95% CI = 0.282–0.326).

## 4. Discussion

This study discusses the impact of vaccination recommendations given by family physicians on influenza and pneumococcal vaccination rates among older adults in China. We found that only 48.78% and 28.56% of older adults had been recommended by their family physician to receive the influenza and pneumococcal vaccines, respectively—an overall low level. However, family physician recommendations had a significant impact on older adults’ vaccination behaviors. Current research on family physician recommendations for influenza and pneumonia vaccination in older adults is almost non-existent. This study has implications for improving influenza and pneumococcal vaccination coverage among the elderly and helps fill a gap in existing research.

Older adults are a key target population for influenza and pneumococcal vaccines, as noted by many vaccination guidelines and studies [32,33,34,35]. The survey shows that the influenza and pneumococcal vaccination rates among older adults were 34.05% and 22.79%, respectively—significantly higher than the national rates reported in 2023 [36]. Possible reasons for this include the increased dissemination of vaccine knowledge and updated vaccination policies. Additionally, the cities in our current survey may have higher levels of economic development than the national average, and rural older adults were not included. Nonetheless, these findings reflect an upward trend in vaccination rates among older adults.

As for the factors of influence, city, educational level, monthly income, health status, and family physician vaccination recommendation all showed significant effects on both influenza and pneumococcal vaccination rates. Previous studies have demonstrated that various factors affect vaccine uptake in older adults, and family physician recommendation is one of the most significant [17,37]. Family physicians’ recommendations play an import role in encouraging vaccination, which can contribute substantially to vaccination uptake [38,39]. In our study, family physician recommendation increased influenza vaccination by 26.3% and pneumococcal vaccination by 23.7%, both with a significant impact. Other studies also suggest that the advice of medical staff such as doctors, physicians and nurses has a strong influence on older adults [40,41]. Trust in physicians makes elderly individuals more likely to follow vaccination recommendations.

We also explored the role of family physician recommendations in subgroups with different characteristics. Some regions in China currently offer free influenza or pneumococcal vaccines, such as Beijing and Shenzhen [42,43]. Our study showed that the free vaccine policy was not statistically significant in predicting overall vaccination rates. However stratified analyses revealed that the policy had a positive effect on both influenza and pneumococcal vaccination among the elderly. While other studies suggest that vaccine price is a factor influencing uptake and that lower prices have a positive effect [44], our findings show that although free vaccination policies provide a small boost, they do not yield statistically significant results in one-way analyses—consistent with past research [45].

In contrast, family physician recommendations produced a much larger effect, highlighting that recommendation behaviors may be more influential than cost. Moreover, vaccination rates were higher when vaccines were both free and recommended by a family physician. This suggests that combining free vaccine policies with increased referrals from medical personnel can more effectively raise vaccination rates among older adults.

Economic level also played a role in influencing vaccination. Our study found that older adults with higher monthly incomes had higher rates of influenza and pneumococcal vaccination, especially those in the CNY 5000–7500 income range [46]. These higher rates in wealthier groups may be linked to greater health awareness, better access to information, or higher willingness to pay.

We also found that family physician recommendations had the greatest boosting effect on vaccination among individuals aged 70–79. These findings highlight the importance of age-specific communication strategies. For example, family physicians may need to take a more proactive and educational approach with the 60–69 age group, emphasizing early protection even if they feel healthy. For the 70–79 age group, leveraging routine visits and reinforcing trust relationships may further increase vaccination uptake. And gender was not an especially impactful factor.

We recommend strengthening the role of medical personnel in promoting influenza and pneumococcal vaccination among older adults. It is worth noting that the implementation of the family physician policy by the Chinese government can improve access to health services and provide older adults with more opportunities to interact with healthcare workers [47]. Family physicians can enhance older adults’ knowledge about influenza and pneumococcal vaccines and encourage positive vaccination behaviors [45]. One study found that how family physicians recommend vaccines also influences the vaccination decisions of older adults [48,49].

Healthcare professionals should adopt different vaccine recommendation strategies based on the characteristics of older adults. For seniors, proactively acquiring vaccine knowledge is a vital health-promoting behavior. Therefore, health education should be further leveraged to promote initiative and awareness about vaccination among the elderly.

This study has some limitations. First, as a cross-sectional study, it cannot infer causality. Second, the study population included only older adults in urban areas and excluded those in rural regions, which may be affected by differences in economic level, education, and access to care. Thirdly, vaccination status was self-reported, and although the survey was conducted face-to-face, there is still potential for information bias. Furthermore, in future research, we could pay attention to detailed information about elderly individuals and family physicians, such as the experience of family physicians. Despite these limitations, this study fills a gap in understanding the role of healthcare workers in promoting vaccination.

## 5. Conclusions

This study analyzed the effect of general family physician recommendations on influenza and pneumococcal vaccination rates among urban Chinese older adults. The results showed that family physician recommendations had a significant effect and that individuals with different demographic characteristics had varying sensitivities to those recommendations. In the future, the role of medical staff in recommending vaccinations should be strengthened, and older adults’ motivation and awareness of necessary vaccination knowledge should be improved.

## Figures and Tables

**Table 1:** Demographic characteristics of participants.

Demographic Characteristics		N	%
City	Beijing	2180	16.31
	Chengdu	2059	15.41
	Hangzhou	2208	16.52
	Qingdao	2140	16.01
	Shenzhen	2253	16.86
	Chongqing	2523	18.88
Gender	Male	6048	45.26
	Female	7315	54.74
Age	60–69	7678	57.46
	70–79	4302	32.19
	≥80	1383	10.35
Ethnicity	Han	13,073	97.83
	Minorities	290	2.17
Education Level	Primary School and Below	3720	27.84
	Junior High School	3992	29.87
	Senior High School	3461	25.90
	Bachelor’s Degree	2050	15.34
	Postgraduate	140	1.05
Monthly Income (CNY *)	≤2500	3850	28.81
	2501–5000	5829	43.62
	5001–7500	2457	18.39
	>7500	1227	9.18
Health Status	Very Poor	215	1.61
	Poor	1188	8.89
	Fair	5367	40.16
	Good	5421	40.57
	Very Good	1172	8.77
Vaccine Price (Influenza Vaccination)	Self-pay	8813	65.95
	Free	4550	34.05
Vaccine Price (Pneumococcal Vaccination)	Self-pay	10,317	77.21
	Free	3046	22.79
Vaccine Uptake (Influenza Vaccination)	No	8813	65.95
	Yes	4550	34.05
Vaccine Uptake (Pneumococcal Vaccination)	No	10,317	77.21
	Yes	3046	22.79
Family Physician Vaccination Recommendation (Influenza Vaccination)	No	6845	51.22
	Yes	6518	48.78
Family Physician Vaccination Recommendation (Pneumococcal Vaccination)	No	9546	71.44
	Yes	3817	28.56

* CNY: Chinese yuan; US dollars (USD) have an average exchange rate of CNY 7.19 per USD in December 2024 [31].

**Table 2:** Factors associated with influenza and pneumococcal vaccination—results of bivariate analyses.

Variable		Influenza Vaccine				Pneumococcal Vaccine			
		Non-Vaccinated	Vaccinated	*p*-Value	χ^2^	Non-Vaccinated	Vaccinated	*p*-Value	χ^2^
		N (%)	N (%)			N (%)	N (%)		
City	Beijing	1472 (67.52%)	708 (32.48%)	<0.001	187.684	1659 (76.10%)	521 (23.90%)	<0.001	271.66
	Chengdu	1414 (68.67%)	645 (31.33%)			1367 (66.39%)	692 (33.61%)		
	Hangzhou	1195 (54.12%)	1013 (45.88%)			1655 (74.95%)	553 (25.05%)		
	Qingdao	1540 (71.96%)	600 (28.04%)			1637 (76.50%)	503 (23.50%)		
	Shenzhen	1470 (65.25%)	783 (34.75%)			1934 (85.84%)	319 (14.16%)		
	Chongqing	1722 (68.25%)	801 (31.75%)			2065 (81.85%)	458 (18.15%)		
Gender	Male	3938 (65.11%)	2110 (34.89%)	0.063	3.458	4635 (76.64%)	1413 (23.36%)	0.154	2.031
	Female	4875 (66.64%)	2440 (33.36%)			5682 (77.68%)	1633 (22.32%)		
Age	60–69	5038 (65.62%)	2640 (34.38%)	1.383	0.501	5929 (77.22%)	1749 (22.78%)	0.318	2.289
	70–79	2846 (66.16%)	1456 (33.84%)			3300 (76.71%)	1002 (23.29%)		
	≥80	929 (67.17%)	454 (32.83%)			1088 (78.67%)	295 (21.33%)		
Ethnicity	Han Chinese	8628 (66.00%)	4445 (34.00%)	0.433	0.007	10,100 (77.26%)	2973 (22.74%)	0.329	0.953
	Minorities	185 (63.79%)	105 (36.21%)			217 (74.83%)	73 (25.17%)		
Education Level	Primary School and Below	2677 (71.96%)	1043 (28.04%)	<0.001	99.695	2972 (79.89%)	748 (20.11%)	<0.001	59.038
	Junior High School	2609 (65.36%)	1383 (34.64%)			3100 (77.66%)	892 (22.34%)		
	Senior High School	2205 (63.71%)	1256 (36.29%)			2680 (77.43%)	781 (22.57%)		
	Bachelor’s Degree	1243 (60.63%)	807 (39.37%)			1472 (71.80%)	578 (28.20%)		
	Postgraduate	79 (56.43%)	61 (43.57%)			93 (66.43%)	47 (33.57%)		
Monthly Income (CNY)	≤2500	2849 (74.00%)	1001 (26.00%)	<0.001	157.152	3095 (80.39%)	755 (19.61%)	<0.001	36.476
	2501—5000	3676 (63.06%)	2153 (36.94%)			4458 (76.48%)	1371 (23.52%)		
	5001—7500	1520 (61.86%)	937 (38.14%)			1824 (74.24%)	633 (25.76%)		
	≥7500	768 (62.59%)	459 (37.41%)			940 (76.61%)	287 (23.39%)		
Health Status	Very Poor	125 (58.14%)	90 (41.86%)	0.004	15.594	132 (61.40%)	83 (38.60%)	<0.001	52.851
	Poor	813 (68.43%)	375 (31.57%)			954 (80.30%)	234 (19.70%)		
	Fair	3599 (67.06%)	1768 (32.94%)			4214 (78.52%)	1153 (21.48%)		
	Good	3519 (64.91%)	1902 (35.09%)			4157 (76.68%)	1264 (23.32%)		
	Very Good	757 (64.59%)	415 (35.41%)			860 (73.38%)	312 (26.62%)		
Family Physician Vaccination Recommendation	No	5405 (78.96%)	1440 (21.04%)	<0.001	1058.091	8145 (85.32%)	1401 (14.68%)	<0.001	1251.479
	Yes	3408 (52.29%)	3110 (47.71%)			2172 (56.90%)	1645 (43.10%)		
Vaccine Price	Self-pay	5871 (66.62%)	2942 (33.38%)	0.476	0.509	4279 (41.48%)	6038 (58.52%)	0.445	0.584
	Free	3059 (67.23%)	1491 (32.77%)			1287 (42.25%)	1759 (57.75%)		

**Table 3:** Vaccine policy differences in the probability of influenza vaccine and pneumococcal vaccine with family physician recommendation and no family physician recommendation.

Family Physician Recommendation	Vaccine Price	Influenza Vaccine							Pneumococcal Vaccine						
		Unadjusted		Adjusted					Unadjusted		Adjusted				
		Vaccinated (%)	*p*-Value	Vaccinated (%) ^a^	Effect of Vaccine Price by Family Physician Recommendation (pp) ^b^	95% CI	Effect of Physician Family Recommendation by Vaccine Price (pp) ^c^	95% CI	Vaccinated (%)	*p*-Value	Vaccinated (%) ^a^	Effect of Vaccine Price by Family Physician Recommendation (pp) ^b^	95% CI	Effect of Family Physician Recommendation by Vaccine Price (pp) ^c^	95% CI
No	Self-pay	18.50%	<0.001	19.89%	reference		reference		14.90%	0.622	14.38%	reference		reference	
	Free	24.40%		21.53%	1.64%	(0.002,0.031) *	reference		14.50%		14.53%	0.15%	(−0.010, 0.011)	reference	
Yes	Self-pay	46.50%	<0.001	47.36%	reference		27.47%	(0.259, 0.290) *	41.60%	0.103	43.16%	reference		28.78%	(0.266, 0.304) *
	Free	51.70%		49.86%	2.50%	(0.003,0.047) *	28.33%	(0.265, 0.301) *	44.30%		43.44%	0.28%	(−0.019, 0.023)	28.91%	(0.267, 0.305) *

* p < 0.05. ^a^ Estimates are from a multivariable logistic regression model adjusted for gender, ethnicity, age, educational level, monthly income, health status. ^b^ Effect of vaccine price by recommendation (pp) represents differences in the probability of vaccination by vaccine price. For instance, among those with no family physician vaccination recommendation, the adjusted influenza vaccine uptake probability of self-paid vaccines was 19.89% and of free vaccines was 21.53%, so the effect of free vaccination in those with no family physician vaccination recommendation was 21.53% − 19.89% = 1.64%. ^c^ The effect of recommendation by vaccine price (pp) represents differences in the probability of vaccination by family physician recommendation. For instance, among self-paid vaccines, uptake with family physician recommendation’s probability was 47.36%; with no family physician recommendation, influenza vaccine probability was 18.50%; the effect of family physician recommendation in self-paid vaccines was 47.36% − 19.89% = 27.47%.

**Table 4:** Age-group differences in the probability of influenza vaccine and pneumococcal vaccine with family physician recommendation and no family physician recommendation.

Family Physician Recommendation	Age	Influenza Vaccine							Pneumococcal Vaccine						
		Unadjusted		Adjusted					Unadjusted		Adjusted				
		Vaccinated (%)	*p*-Value	Vaccinated (%) ^a^	Effect of Age by Family Physician Recommendation (pp) ^b^	95% CI	Effect of Family Physician Recommendation by Age (pp) ^c^	95% CI	Vaccinated (%)	*p*-Value	Vaccinated (%) ^a^	Effect of Age by Family Physician Recommendation (pp) ^b^	95% CI	Effect of Family Physician Recommendation by Age (pp) ^c^	95% CI
No	60–69	19.70%	0.004	21.37%	reference		reference		14.20%	0.025	14.12%	reference		reference	
	70–79	23.30%		22.28%	0.91%	(−0.005,0.024)	reference		16.00%		15.41%	1.29%	(0.001,0.025) *	reference	
	≥80	20.90%		20.47%	−0.90%	(−0.030,0.013)	reference		13.10%		13.93%	−0.19%	(−0.020,0.016)	reference	
Yes	60–69	49.30%	0.004	49.48%	reference		28.11%	(0.267, 0.300) *	43.60%	0.643	42.68%	reference		28.56%	(0.267, 0.304) *
	70–79	46.30%		50.81%	1.33%	(−0.008,0.034)	28.53%	(0.270, 0.305) *	42.70%		45.21%	2.53%	(0.002,0.049) *	29.80%	(0.277, 0.317) *
	≥80	43.30%		48.12%	−1.36%	(−0.046,0.019)	27.65%	(0.260, 0.298) *	41.30%		42.30%	−0.38%	(0.033,0.838) *	28.37%	(0.277, 0.317) *

* *p* < 0.05. ^a^ Estimates are from a multivariable logistic regression model adjusted for gender, ethnicity, monthly income, educational level, health status, vaccine price. ^b^ Effect of age by recommendation (pp) represents differences in the probability of vaccination by age. For instance, among those with no family physician vaccination recommendation, the adjusted influenza vaccine uptake probability of 70–79-year-olds was 22.28%; the probability of 60–69% was 21.37%, so the effect of age (70–79 years old) on influenza vaccine uptake in those with no family physician vaccination recommendation was 22.28% − 21.37% = 0.91%. ^c^ The effect of recommendation by age (pp) represents differences in the probability of vaccination by family physician recommendation. For instance, among 60–69-year-olds with family physician recommendation, influenza vaccine probability was 49.48%, while the probability for those with no family physician recommendation was 21.37%; the effect of family physician recommendation in 60–69-year-olds was 49.48% − 21.37% = 28.11%.

**Table 5:** Gender differences in the probability of influenza vaccine and pneumococcal vaccine with family physician recommendation and no family physician recommendation.

Family Physician Recommendation	Gender	Influenza Vaccine							Pneumococcal Vaccine						
		Unadjusted		Adjusted					Unadjusted		Adjusted				
		Vaccinated (%)	*p*-Value	Vaccinated (%) ^a^	Effect of Gender by Family Physician Recommendation (pp) ^b^	95% CI	Effect of Family Physician Recommendation by Gender (pp) ^c^	95% CI	Vaccinated (%)	*p*-Value	Vaccinated (%) ^a^	Effect of Gender by Family Physician Recommendation (pp) ^b^	95% CI	Effect of Family Physician Recommendation by Gender (pp) ^c^	95% CI
No	Male	20.30%	0.188	19.72%	reference		reference		14.50%	0.651	14.48%	reference		reference	
	Female	21.60%		20.02%	0.30%	(−0.009, 0.015)	reference		14.80%		14.56%	0.08%	(−0.010, 0.012)	reference	
Yes	Male	49.10%	0.029	47.11%	reference		27.39%	(0.262, 0.296) *	44.40%	0.114	43.36%	reference		28.88%	(0.270, 0.306) *
	Female	46.40%		47.57%	0.46%	(−0.014, 0.024)	27.55%	(0.264, 0.298) *	41.90%		43.51%	0.15%	(−0.020, 0.023)	28.95%	(0.271, 0.307) *

* *p* < 0.05. ^a^ Estimates are from a multivariable logistic regression model adjusted for ethnicity, age, educational level, health status, vaccine price. ^b^ Effect of gender by recommendation (pp) represents differences in the probability of vaccination by monthly income. For instance, among those with no family physician vaccination recommendation, the adjusted influenza vaccine uptake probability of males was 19.72% and the probability of females was 20.02%, so the effect of gender in those no family physician vaccination recommendation was 20.02% − 19.72% = 0.30%. ^c^ The effect of recommendation by gender (pp) represents differences in the probability of vaccination by family physician recommendation. For instance, among males with family physician recommendations, influenza vaccine probability was 47.11%, while males with no family physician recommendation had a probability of 19,72%; the effect of family physician recommendation in males was 47.11% − 19.72% = 27.39%.

**Table 6:** Monthly income differences in the probability of influenza vaccine and pneumococcal vaccine with family physician recommendation and no family physician recommendation.

Family Physician Recommendation	Monthly Income (CNY)	Influenza Vaccine							Pneumococcal Vaccine						
		Unadjusted		Adjusted					Unadjusted		Adjusted				
		Vaccinated (%)	*p*-Value	Vaccinated (%) ^a^	Effect of Monthly Income by Family Physician Recommendation (pp) ^b^	95% CI	Effect of Family Physician Recommendation by Monthly Income (pp) ^c^	95% CI	Vaccinated (%)	*p*-Value	Vaccinated (%) ^a^	Effect of Monthly Income by Family Physician Recommendation (pp) ^b^	95% CI	Effect of Family Physician Recommendation by Monthly Income (pp) ^c^	95% CI
No	<2500	13.70%	<0.001	15.83%	reference		reference		13.23%	<0.001	12.25%	reference		reference	
	2500–5000	22.10%		21.37%	5.54%	(0.041, 0.070) *	reference		14.06%		14.41%	2.16%	(0.010,0.034) *	reference	
	5000–7500	25.70%		22.36%	6.53%	(0.044, 0.087) *	reference		16.09%		18.08%	5.83%	(0.042,0.075) *	reference	
	>7500	29.30%		20.16%	4.33%	(0.016, 0.070) *	reference		13.28%		16.23%	3.98%	(0.019,0.061) *	reference	
Yes	<2500	40.00%	<0.001	40.39%	reference		24.56%	(0.232, 0.266) *	40.92%	<0.001	38.54%	reference		27.69%	(0.256, 0.297) *
	2500–5000	50.10%		49.48%	9.09%	(0.067, 0.115) *	28.11%	(0.267, 0.300) *	42.62%		43.06%	4.52%	(0.020,0.070) *	28.56%	(0.267, 0.304) *
	5000–7500	54.00%		50.92%	10.53%	(0.072, 0.139) *	28.56%	(0.270, 0.306) *	46.55%		49.79%	11.25%	(0.082,0.144) *	30.46%	(0.282, 0.326) *
	>7500	47.20%		47.63%	7.24%	(0.029, 0.116) *	27.47%	(0.258, 0.297) *	41.01%		46.53%	7.99%	(0.040,0.120) *	27.73%	(0.250, 0.304) *

* *p* < 0.05. ^a^ Estimates are from a multivariable logistic regression model adjusted for gender, ethnicity, age, educational level, health status, vaccine price. ^b^ Effect of monthly income by recommendation (pp) represents differences in the probability of vaccination by monthly income. For instance, among those with no family physician vaccination recommendation, the adjusted influenza vaccine uptake probability of CNY <2500 was 15.83% and the probability of CNY 2500–5000 was 21.37%, so the effect of monthly income (CNY 2500–5000) in those with no family physician vaccination recommendation was 21.37% − 15.83% = 5.54%. ^c^ The effect of recommendation by monthly income (pp) represents differences in the probability of vaccination by physician recommendation. For instance, among those whose monthly income was CNY 2500–5000, those with family physician recommendations had an influenza vaccine probability of 49.48%, while those with no family physician recommendation had a probability of 21.37%; the effect of family physician recommendation in those with CNY 2500–5000 monthly income was 49.48% − 21.37% = 28.11%.

## Data Availability

The data within this article will be shared upon reasonable request to the corresponding author.

## Supplementary Materials

The following supporting information can be downloaded at: https://www.mdpi.com/article/10.3390/vaccines13080886/s1, File S1: Questionnaire Structure and Content.

## References

[1] Voordouw B.C., van der Linden P.D., Simonian S., van der Lei J., Sturkenboom M.C., Stricker B.H. (2003). Influenza vaccination in community-dwelling elderly: Impact on mortality and influenza-associated morbidity. Arch. Intern. Med..

[2] Rose N., Storch J., Mikolajetz A., Lehmann T., Reinhart K., Pletz M.W., Forstner C., Vollmar H.C., Freytag A., Fleischmann-Struzek C. (2021). Preventive effects of influenza and pneumococcal vaccination in the elderly—Results from a population-based retrospective cohort study. Hum. Vaccines Immunother..

[3] Dorrington M.G., Bowdish D.M.E. (2013). Immunosenescence and novel vaccination strategies for the elderly. Front. Immunol..

[4] (2019). Global Influenza Strategy 2019–2030[EB/OL]. https://www.who.int/publications/i/item/9789241515320.

[5] Al-Jumaili A., Dawood H.N., Ikram D., Al-Jabban A. (2023). Pneumococcal disease: Global disease prevention strategies with a focus on the challenges in iraq. Int. J. Gen. Med..

[6] (2024). Pneumococcal Disease Surveillance and Trends|Pneumococcal|CDC[EB/OL]. https://www.cdc.gov/pneumococcal/php/surveillance/index.html.

[7] Zhou M., Zhan J., Kong N., Campy K.S., Chen Y. (2022). Factors associated with intention to uptake pneumococcal vaccines among Chinese elderly aged 60 years and older during the early stage of COVID-19 pandemic. Psychol. Health Med..

[8] Yunhua B., Peng B., Shuping L., Zheng Z. (2022). A narrative review on vaccination rate and factors associated with the willingness to receive pneumococcal vaccine in Chinese adult population. Hum. Vaccines Immunother..

[9] Mameli C., Cocchi I., Fumagalli M., Zuccotti G. (2019). Influenza vaccination: Effectiveness, indications, and limits in the pediatric population. Front. Pediatr..

[10] (2024). WHO Recommendations for Routine Immunization—Summary Tables [EB/OL]. https://www.who.int/teams/immunization-vaccines-and-biologicals/policies/who-recommendations-for-routine-immunization---summary-tables.

[11] Theidel U., Kuhlmann A., Braem A. (2013). Pneumococcal vaccination rates in adults in germany: An analysis of statutory health insurance data on more than 850,000 individuals. Dtsch. Arztebl. Int..

[12] (2024). Flu Vaccination Coverage, United States, 2020–2021 Influenza Season|FluVaxView|CDC[EB/OL]. https://www.cdc.gov/fluvaxview/coverage-by-season/2020-2021.html.

[13] (2025). Surveillance of Influenza and Other Seasonal Respiratory Viruses in the UK, Winter 2022 to 2023[EB/OL]. https://www.gov.uk/government/statistics/annual-flu-reports/surveillance-of-influenza-and-other-seasonal-respiratory-viruses-in-the-uk-winter-2022-to-2023.

[14] Kohlhammer Y., Schnoor M., Schwartz M., Raspe H., Schäfer T. (2007). Determinants of influenza and pneumococcal vaccination in elderly people: A systematic review. Public Health.

[15] Conlon A., Ashur C., Washer L., Hofmann Bowman M. (2021). Impact of the influenza vaccine on COVID-19 infection rates and severity. Am. J. Infect. Control.

[16] Candelli M., Pignataro G., Gullì A., Nista E., Petrucci M., Saviano A., Marchesini D., Covino M., Ojetti V. (2021). Effect of influenza vaccine on COVID-19 mortality: A retrospective study. Intern. Emerg. Med..

[17] Lai X., Lyu Y., Zhang H., Fang H. (2022). PPSV-23 recommendation and vaccination coverage in China: A cross-sectional survey among healthcare workers, older adults and chronic disease patients. Expert Rev. Vaccines.

[18] Yang J., Atkins K.E., Feng L., Pang M., Zheng Y., Liu X., Cowling B.J., Yu H. (2016). Seasonal influenza vaccination in China: Landscape of diverse regional reimbursement policy, and budget impact analysis. Vaccine.

[19] Pan Y., Wang Q., Yang P., Wang Q., Peng Y., Li Z., Wu S., Yi Z., Ying S., Wei D. (2017). Influenza vaccination in preventing outbreaks in schools: A long-term ecological overview. Vaccine.

[20] Principi N., Camilloni B., Esposito S. (2018). Influenza immunization policies: Which could be the main reasons for differences among countries?. Hum. Vaccines Immunother..

[21] Bazargan M., Martinez-Hollingsworth A., Cobb S., Kibe L.W. (2022). Correlates of influenza vaccination among underserved Latinx middle-aged and older adults: A cross-sectional survey. BMC Public Health.

[22] Chen H., Li Q., Zhang M., Gu Z., Zhou X., Cao H., Wu F., Liang M., Zheng L., Xian J. (2022). Factors associated with influenza vaccination coverage and willingness in the elderly with chronic diseases in Shenzhen, China. Hum. Vaccines Immunother..

[23] Yan S., Wang Y., Zhu W., Zhang L., Gu H., Liu D., Zhu A., Xu H., Hao L., Ye C. (2021). Barriers to influenza vaccination among different populations in shanghai. Hum. Vaccines Immunother..

[24] Yu M., Yao X., Liu G., Wu J., Lv M., Pang Y., Xie Z., Huang Y. (2022). Barriers and facilitators to uptake and promotion of influenza vaccination among health care workers in the community in Beijing, China: A qualitative study. Vaccine.

[25] National Health Commission, National Administration of Traditional Chinese Medicine (2018). Guiding Opinions on Regulating the Management of Family Doctor Contracted Services (Document No. 35 [2018]). https://www.gov.cn/zhengce/zhengceku/2018-12/31/content_5435461.htm.

[26] Lu P.J., Srivastav A., Amaya A., Dever J., Roycroft J., Kurtz M., O’Halloran A., Williams W. (2018). Association of provider recommendation and offer and influenza vaccination among adults aged ≥18 years—United states. Vaccine.

[27] Tucker J.D., Cheng Y., Wong B., Gong N., Nie J.B., Zhu W., McLaughlin M.M., Xie R., Deng Y., Huang M. (2015). Patient-physician mistrust and violence against physicians in Guangdong province, China: A qualitative study. BMJ Open.

[28] Lu M., Li Z., Niu Q., Wang C., Ma Y., Wang Y., Zhang M., Bai Y., Ou L., Zhang Y. (2025). Analysis of the vaccination status of three non-immunization program vaccines among people aged 50 and above in Henan Province, 2020–2023. Mod. Prev. Med..

[29] Liu S., Xu E., Liu Y., Xu Y., Wang J., Du J., Zhang X., Che X., Gu W. (2014). Factors associated with pneumococcal vaccination among an urban elderly population in China. Hum. Vaccines Immunother..

[30] Mui L.W.H., Chan A.Y.S., Lee A. (2013). Cross-sectional study on attitudes among general practitioners towards pneumococcal vaccination for middle-aged and elderly population in Hong Kong. PLoS ONE.

[31] State Administration of Foreign Exchange [EB/OL]. https://www.safe.gov.cn/safe/rmbhlzjj/index.html.

[32] (2019). Pneumococcal Polysaccharide Vaccine (PPSV23)—What You Need to Know: MedlinePlus Medical Encyclopedia[EB/OL]. https://medlineplus.gov/ency/article/007607.htm.

[33] The Weekly Epidemiological Record (WER)[EB/OL]. https://www.who.int/publications/journals/weekly-epidemiological-record.

[34] Australian Government Department of Health and Aged Care (2019). Influenza Infection (flu)—CDNA National Guidelines for Public Health Units [EB].

[35] Chinese Center for Disease Control and Prevention (2024). Technical guidelines for influenza vaccination in China (2023–2024). Chin. J. Viral Dis..

[36] Cheng L., Li L., Cao L., Song Y., Zhang Z., Yin Z. (2024). Analysis of the vaccination status of three non-immunization program vaccines among people aged ≥60 years in China, 2019–2023. Chin. J. Prev. Med..

[37] Al Rifai M., Khalid U., Misra A., Liu J., Nasir K., Cainzos-Achirica M., Mahtta D., Ballantyne C., Petersen L., Virani S. (2021). Racial and geographic disparities in influenza vaccination in the U.S. among individuals with atherosclerotic cardiovascular disease: Renewed importance in the setting of COVID-19. Am. J. Prev. Cardiol..

[38] Campos-Outcalt D., Jeffcott-Pera M., Carter-Smith P., Schoof B.K., Young H.F. (2010). Vaccines provided by family physicians. Ann. Fam. Med..

[39] Balakrishnan V.S. (2015). Physicians’ recommendation affects HPV vaccination uptake. Lancet Oncol..

[40] Ridda I., Motbey C., Lam L., Lindley I.R., McIntyre P.B., MacIntyre C.R. (2008). Factors associated with pneumococcal immunisation among hospitalised elderly persons: A survey of patient’s perception, attitude, and knowledge. Vaccine.

[41] Sakamoto A., Chanyasanha C., Sujirarat D., Matsumoto N., Nakazato M. (2018). Factors associated with pneumococcal vaccination in elderly people: A cross-sectional study among elderly club members in Miyakonojo city, Japan. BMC Public Health.

[42] (2024). Launch of Influenza Vaccination in the City. [EB/OL]. https://www.beijing.gov.cn/fuwu/bmfw/sy/jrts/202409/t20240913_3865950.html.

[43] Shenzhen Municipal Health Commission (2023). [EB/OL]. https://wjw.sz.gov.cn/ztzl/ymjz/xgzc/content/post_10938913.html.

[44] de Boer P.T., van Werkhoven C.H., van Hoek A.J., Knol M.J., Sanders E.M., Wallinga J., de Melker H.E., Steens A. (2024). Higher-valency pneumococcal conjugate vaccines in older adults, taking into account indirect effects from childhood vaccination: A cost-effectiveness study for the NETHERLANDS. BMC Med..

[45] Minghuang J., Pengchao L., Xuelin Y., Khezar H., Yilin G., Shan Z., Jin P., Xinke S., Zhaojing P., Yifan H. (2021). Preference of influenza vaccination among the elderly population in Shaanxi province, China. Hum. Vaccines Immunother..

[46] Alharthi M.S., Alshehri A.A., Baali F.H., Gong Y., Zhu S., Peng J., Shi X., Pu Z., Huang Y., Fang Y. (2025). Public perceptions and influencing factors of seasonal influenza vaccine uptake in Makkah region, Saudi Arabia: A cross-sectional study. Front. Public Health.

[47] (2022). Guiding Opinions on Promoting the High-Quality Development of Family Doctor Contracted Services. https://www.gov.cn/zhengce/zhengceku/2022-03/15/content_5679177.htm.

[48] Briggs L., Fronek P., Quinn V., Wilde T. (2019). Perceptions of influenza and pneumococcal vaccine uptake by older persons in Australia. Vaccine.

[49] Hou J., Michaud C., Li Z., Dong Z., Sun B., Zhang J., Cao D., Wan X., Zeng C., Wei B. (2014). Transformation of the education of health professionals in China: Progress and challenges. Lancet.

