# Supplementary material for: Do Family Physicians’ Recommendations for Influenza and Pneumococcal Vaccines Impact the Elderly Aged ≥60 Years? A Cross-Sectional Study in Six Chinese Cities
Source: Vaccines (Basel). 2025 Aug 21;13(8):886. doi: 10.3390/vaccines13080886 (PMC12389752; doi:10.3390/vaccines13080886)
Supplement: Supplementary file 1 [file vaccines-13-00886-s001.zip › vaccines-3760025-supplementary.pdf]

File S1: Questionnaire Structure and Content

- **Informed consent statement**

Hello. This questionnaire is organized and implemented by the *Peking Union Medical College, Chinese Academy of Medical Sciences*, aiming to understand the vaccination behavior and intention of the elderly, and we hope that you can answer truthfully according to your own situation, and we thank you for your understanding and support. Your answers and participation will help us to better develop and optimize vaccine education programs. All information collected about you in the questionnaire will be used for the research of this evaluation survey and will not be disclosed to a third party or used for purposes other than this evaluation without your consent.

If you are willing to participate in this survey, please tick and sign your name.

I agree to participate in the survey ☐

Please sign your name:_________

- **First section:basic information**

**Questions**	**Type**
Q1. Gender: (1)Male (2)Female	Multiple choice
Q2. Date of birth:	Fill in the blanks
Q3. Ethnicity: (1) Han (2)Minorities	Multiple choice
Q4. Which of the following best describes your education level:<br>(1)Primary school and below<br>(2)Junior high school<br>(3)Senior high school<br>(4)Bachelor's Degree<br>(5)Postgraduate	Multiple choice
Q5.Which of the following best describes your monthly income?(CNY)<br>(1)≤2500<br>(2)2501-5000<br>(3)5001-7500<br>(4)>7500	Multiple choice
Q6. Which of the following best describes your health status over the past year?<br>(1)Very poor<br>(2)poor<br>(3)Fair<br>(4)Good<br>(5)Very good	Multiple choice
Q7.Is the influenza vaccine you received free of charge?<br>(1)Yes (2)No	Multiple choice
Q8.Is the influenza vaccine you received free of charge?<br>(1)Yes (2)No	Multiple choice

- **Second section: Influenza and Pneumococcal Vaccination Status**

Questions	Type
Q1.Have you received influenza vaccine immunizations for adults in the past one year?<br>(1)Yes (2)No	Multiple choice
Q2.Have you ever received pneumococcal vaccine immunizations for adults?<br>(1)Yes (2)No	Multiple choice
Q3.Has a family physician ever recommended that you receive the influenza vaccine?<br>(1)Yes (2)No	Multiple choice
Q4.Has a family physician ever recommended that you receive the pneumococcal vaccination?<br>(1)Yes (2)No	Multiple choice
Q5.What are your reasons for getting influenza vaccinated?	Multiple choice
Q6.What are your reasons for getting pneumococcal vaccinated?	Multiple choice
Q7.What are your reasons for not getting influenza vaccinated?	Multiple choice
Q8.What are your reasons for not getting pneumococcal vaccinated?	Multiple choice
... ...	

- **Third section: Vaccine hesitancy toward influenza, pneumonia vaccinations**

This section is measured using 5Cs hesitancy scale, include confidence, complacency, constraints, calculation, collective responsibility.
